# Improving retention of pediatric feeding tubes with a nasal bridle: a randomized controlled trial

**DOI:** 10.1186/s13063-025-08867-x

**Published:** 2025-05-14

**Authors:** Megan Foster, Veronica Armijo-Garcia, Jonathan Gelfond, Andrew D. Meyer

**Affiliations:** 1 Division of Critical Care, Department of Pediatrics, Long School of Medicine at the University of Texas Health Science Center, 7703 Floyd Curl Drive, MC 7829, San Antonio, TX 78229 USA; 2 Department of Epidemiology and Biostatistics, Long School of Medicine at the University of Texas Health Science Center, San Antonio, TX USA

**Keywords:** Feeding tube, Nasoenteral feeding tube, Dislodgement, Nasal bridle

## Abstract

**Background:**

Nasoenteric feeding tubes are necessary in hospitalized children to deliver nutrition and medication. Traditionally, adhesive tape secures these feeding tubes but fails to prevent 40% of tube dislodgements. The nasal bridle, a thin plastic anchor placed around the vomer bone, is an increasingly used method for tube securement. Our objective is to compare AMT Bridle Pro® nasal bridle versus conventional tape to safely reduce tube dislodgement in pediatric patients.

**Methods:**

A prospective, open-label randomized controlled trial was carried out between February 2020 and January 2021 at a tertiary pediatric hospital. Infants, children, and adolescents less than 18 years of age with an order to place a nasogastric or post-pyloric feeding tube were approached for enrollment. Exclusion criteria included facial trauma, nasal airway obstruction, or thrombocytopenia. After obtaining consent, patients were randomized to AMT Bridle Pro® nasal bridle or conventional tape to secure the feeding tube. The primary outcome was the frequency of feeding tube dislodgement, defined as unintentional tube removal or change in position. Secondary outcomes included days to feeding tube dislodgement, number of dislodgements per 10 tube days, resource use, and complications from tube securement.

**Results:**

A total of 35 patients were randomized and equally split to the bridle (*n* = 17) and tape arm (*n* = 18). The primary analysis revealed the rate of feeding tube dislodgement over 30 days was significantly higher in the tape group compared to the bridle group with an attributable risk reduction of 57% (hazard ratio = 6.3, 95% CI 2.4–16.5, *p* < 0.001). After 30 days, tubes dislodged at a proportion 88% (15) in the tape arm compared to 31% (5) in the bridle arm (risk ratio = 2.82; 95% CI: 1.34–5.96; *p* = 0.001). There were no serious adverse events. Four patients in the tape group developed erythema and skin breakdown where the tube was secured with tape. One patient was withdrawn from the bridle group because they developed erythema on the nasal septum after placement, which resolved quickly upon removal of the bridle.

**Conclusion:**

Securing nasoenteric feeding tubes with the AMT Bridle Pro® can effectively reduce tube dislodgements in hospitalized children.

**Trial registration:**

ClinicalTrials.gov NCT04621734. Registered on November 3, 2020. https://clinicaltrials.gov/search?cond=NCT04621734.

**Supplementary Information:**

The online version contains supplementary material available at 10.1186/s13063-025-08867-x.

## Background

Hospitalized children are at increased risk for malnutrition and can have one or more barriers to tolerating oral nutrition and medication [1]. Enteral nutrition has many therapeutic benefits for children including improved wound healing, utilization of gastric organs, and improved maintenance of growth [1]. Studies document that early parenteral nutrition increases the risk of hospital-associated infectious and noninfectious complications [2]. To ensure enteral nutrition, pediatricians will commonly use nasogastric feeding tubes (NGT) to deliver nutrition and medications. Traditionally, these tubes are secured with adhesive tape to the nasal bridge or face. However, unintentional tube dislodgement due to patients pulling on the tube or movement during patient transfer and repositioning continues to be a common occurrence [3]. Adult literature has documented tube dislodgement rates from 30 to 70% and pediatric studies document dislodgement rates as high as 40% although this may be underreported [4, 5]. An early study in pediatric double staged laryngotracheoplasty patients documented that the bridle successfully reduced dislodgements, radiology exposure, and length of stay [6]. This was further confirmed by Lavoie et al. in their retrospective study of 755 children that those with bridled NGTs were 16.67 times less likely to experience one or more dislodgments, 2.5 times less likely to have one more emergency department (ED) visit, 4.76 times less likely to require one more radiographic exposure, and a shorter hospital stay (28 vs. 54 days) than unbridled children (all *p* values < 0.02) [7]. These studies suggest that controlled research is needed to determine the effect that bridles must increase the number of children being fed at home, reduction in health care costs, and providing important nutrition for growth. Moreover, frequent replacement of a feeding tube increases the risk to patient safety by increasing the chance of an improper placement to the lung or causing a perforation [8]. Lastly, repeat tube placement consumes hospital resources from the cost of replacement tubes, radiographs, and staff hours spent on these procedures [9].

**Fig. 1:**
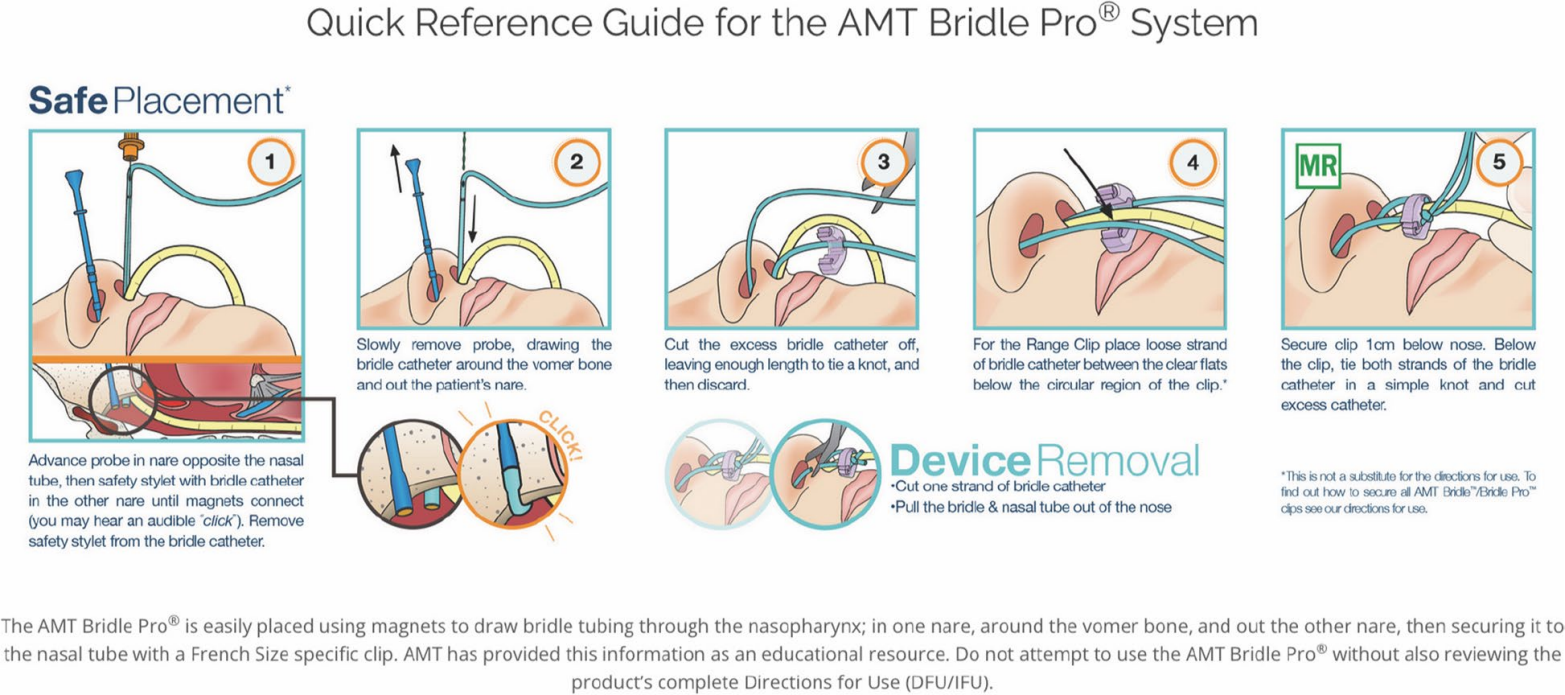
BridlePro™ insertion procedure from the AMT website. Permission obtained from the manufacturer to publish

To reduce complications from traditional tape methods, healthcare professionals need a safe, inexpensive, and effective device to improve retention of nasoenteric feeding tubes. Over 40 years, the nasal bridle device has been improved. The Applied Medical Technology, Inc. (AMT) Bridle Pro® uses two soft but sturdy probes with magnetic tips to facilitate placement of the bridle tubing around the vomer bone (Fig. 1). Although several companies manufacture a bridle, AMT designed, and Food and Drug Administration (FDA) cleared a device for both pediatric and adult patients (Brecksville, OH, USA). Each Bridle Pro® Range Clip can accommodate 2–3 sizes of feeding tubes, ranging from neonatal size bridles (5–6 Fr) to adult size bridles (16–18 Fr). With this product line, AMT also introduced a monofilament tubing that is softer, smoother, and easier to clean as compared to umbilical tape.

Several studies in adult patients documented significant reduction in tube dislodgement by 25–60% when a nasal bridle was used to secure the feeding tube [2–4]. However, only a few retrospective cohort studies in pediatrics have documented success with a nasal bridle [6, 7]. McBride et al. documented that the bridle was not only successful in the hospital but also facilitated early discharge to home [10]. As biomedical devices require only safety approval from the FDA, independent controlled trials are necessary to define their efficacy in patient outcomes. One previous randomized trial comparing the AMT Bridle Pro® to tape securement in pediatric rehabilitation patients but experienced significant difficulty in standardized recruitment and randomization resulting in ineffective power [11]. Our objective is to conduct a single site randomized controlled pediatric trial that compares the efficacy of the AMT Bridle Pro® vs. tape for nasogastric tube securement. Our hypothesis is that the AMT Bridle Pro® nasal bridle will significantly reduce dislodgement of pediatric feeding tubes compared to traditional adhesive methods. Success of this repeat study may inform the development of large, multicenter, pediatric randomized controlled trial of the AMT Bridle Pro® to evaluate if it can improve patient-related outcomes of early nutrition, satisfaction, anxiety, and cost.

## Methods

### Study design

This was a prospective, open-label, randomized control trial conducted in the inpatient pediatric population at our large county hospital in South Texas. Our inpatient pediatric care area includes the pediatric intensive care unit (PICU), pediatric congenital cardiac unit (PCCU), and the pediatric acute care units. The trial was approved by the University of Texas Health Science Center Institutional Review Board (UTHSCSA IRB# 20190225H) and retrospectively registered at ClinicalTrials.gov (NCT04621734). Written informed consent was obtained from all participants prior to enrollment. Assent was obtained from participants if they were over the age of 7 at time of parental consent.

### Participants

Inclusion criteria was children between birth and 18 years of age, admitted to an inpatient pediatric floor, and have an order from the clinical team to place a nasogastric or post-pyloric feeding tube. Exclusion criteria include facial trauma, nasal airway obstruction, history of septoplasty or vomer bone graft, or thrombocytopenia (< 100k).

### Randomization and blinding

After consent and assent were obtained, patients were randomized into the tape arm or the bridle arm by the Research Electronic Data Capture (REDCap) randomization tool using a ratio of 1:1 block randomization of 10. The randomization sequence was managed by an independent statistician and concealed from investigators through sealed, opaque envelopes. As this was an open-label study, both participants and investigators were aware of the treatment allocation.

### Interventions

After randomization, the bedside nurse placed a feeding tube according to standard nursing practice with a routine follow-up radiograph confirming proper tube placement (nasogastric or post-pyloric positioning determined by the primary team). If the patient was randomized to the tape group, the tube was secured by using conventional taping methods commonly practiced in our institution. While no standardized taping protocol was enforced, techniques typically involved anchoring the tube to the patients nose or cheek using hypoallergenic adhesive tape. If the patient was randomized to the bridle group, the bridle was placed, and the tube was secured by a member of the study team who were trained in bridle placement. For bridle placement, there were no added sedative medications or restraint procedures used outside of the standard of care for feeding tube placement in pediatric patients. For this study, AMT supplied the 5–6 Fr and 8–10 Fr bridles to which are appropriate for most pediatric patients.

### Outcomes

Patients were followed for 30 days because the Bridle Pro® is FDA cleared for 30 days of continuous use. The study ended if the patient reached 30 days after enrollment, or the patient no longer required the feeding tube as determined by the primary team, or the feeding tube was dislodged. Feeding tube dislodgement was defined as complete dislodgement of the tube from the patient, any change in tube position according to external depth indicators on the tube, or any radiographic evidence of change in position from intended location. Clinical data was collected from the electronic health record and entered a study database using REDCap. Data collected included age, race, gender, location of admission, reason for admission, and hospital length of stay (Table 1). Primary outcome was the frequency of feeding tube dislodgement. Secondary outcomes included days to feeding tube dislodgement, PICU and hospital length of stay, time missed nutrition (min) from dislodged tubes, number of X-rays to confirm placement of dislodged tubes, and complications from tube securement.

**Table 1:** Demographics of study population

Characteristic	Bridle (***n*** = 16)	Tape (***n*** = 17)
Age, years (min, max)	6.5 (0.1, 17.1)	9.1 (0.1, 17.7)
Male, ***n*** (%)	12 (75)	10 (58)
Weight, kg (min, max)	35 (4, 111)	41 (3.5, 110)
Post-pyloric, ***n*** (%)	9 (56)	6 (35)
Intubated, ***n*** (%)^a^	10 (63)	5 (29)
Reason for admission (***n***)		
Trauma	9	6
Medical	4	6
Congenital heart disease	3	5

^a^The intubated percentage indicates the percentage of patients intubated at the time of feeding tube placement

### Samples size

The primary hypothesis was that the rate of dislodgment would be less with the bridle compared to the tape. Our power analysis was based on a log rank test with a sample size of 30 in each of the two arms (60 total) to achieve 91% power (two-sided alpha = 0.05) assuming a 90% dislodgement rate within 10 days in the control arm and a corresponding 60% dislodgement rate in the bridle arm. An interim analysis for efficacy and safety was performed after 50% enrollment of the planned participants. The proposed study timeline was 18 to 24 months.

### Statistical analysis

Comparisons of demographic continuous variables were analyzed by means and standard errors and categorical variables were analyzed by percentages. To compare the number of dislodged and additional films (> 1) needed confirm placement, we use a two-tailed Fisher’s exact test and the Koopman asymptomatic unpaired score to determine the risk ratios and their confidence intervals. Kaplan–Meier survival curve was constructed to demonstrate time to tube dislodgement between the two groups. The primary analysis was a Mantel–Haenszel hazard model testing the effect of the tape compared to bridle while adjusting for age and gender. All other analysis used unadjusted data. For the other length of stay outcomes, the data was log-transformed and compared with a Welch unequal-variance *t*-test and then back-transformed to display geometric means and CIs. Due to small sample size collected for missed nutrition data, a Mann–Whitney *U* test was performed reporting median difference and CI. Tests of significance were performed using a *p* value of 0.05. Data collection was conducted through REDCap and analysis conducted using Jamovi for Mac OS (v1.6.23.0), GraphPad Prism 10 for Mac OS (v10.4.2), and R (v4.0, Vienna, Austria).

## Results

**Fig. 2:**
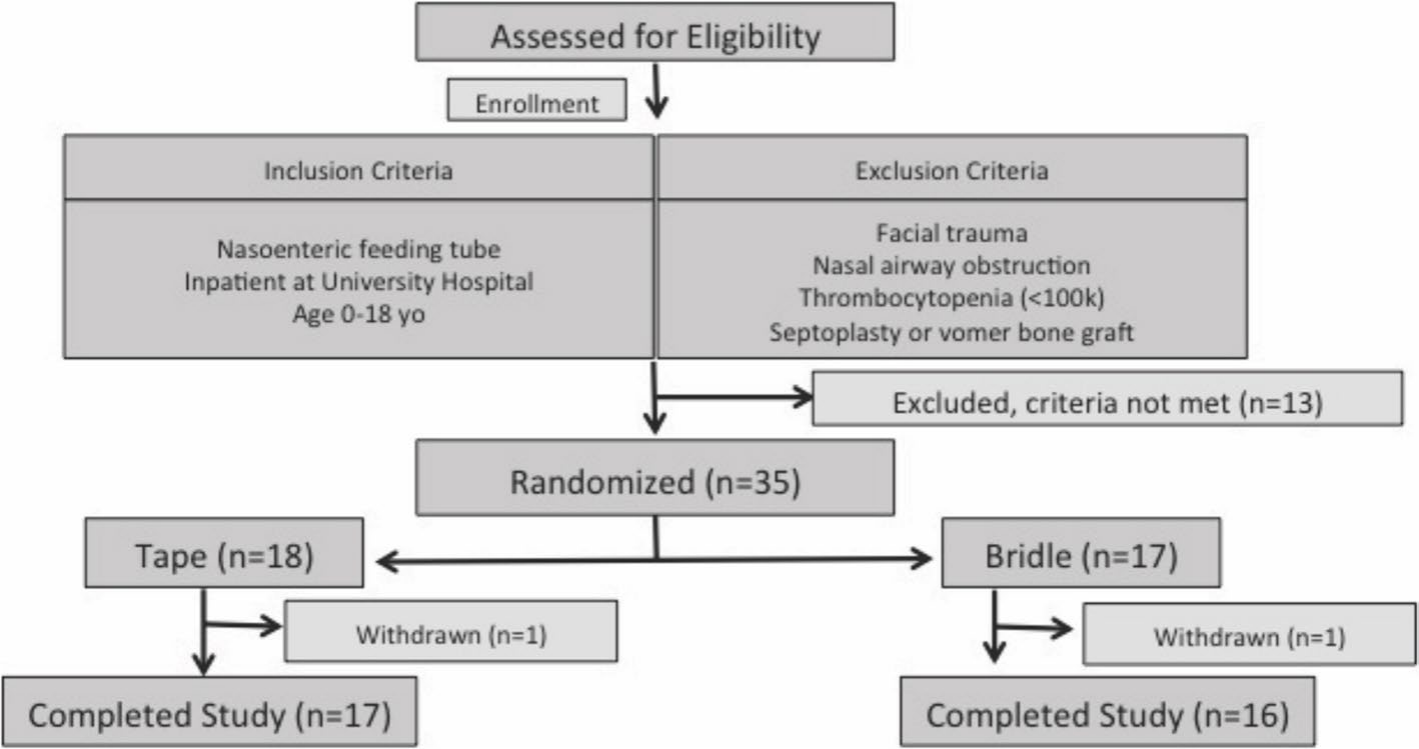
Enrollment and randomization flowchart

From February 2020 through January 2021, 48 patients were screened for enrollment of which 35 met the eligibility criteria and were randomized into either the bridle group (*n* = 17) or the tape group (*n* = 18) (Fig. 2). Two patients withdrew from the study. One from the bridle group for a presumed allergic reaction to the monofilament tubing that resolved 24 h after removal. One from the tape group because they transferred to another institution before completing the study. Interim analysis of the data documented change in the primary outcome, so the study enrollment was completed early. The bridle group had an average age of 6.5 years (min 0.1, max 17.1) and average weight of 35 kg (min 4, max 111). The tape group had an average age of 9.1 years (min 0.1, max 17.7) and average weight of 41 kg (min 3.5, max 110). There was no significant difference in age, gender, weight, incidence of post-pyloric tube placement, or intubation status between the groups. Cohort demographics are reported in Table 1. Most of the patients were PICU admissions following a traumatic injury.

**Fig. 3:**
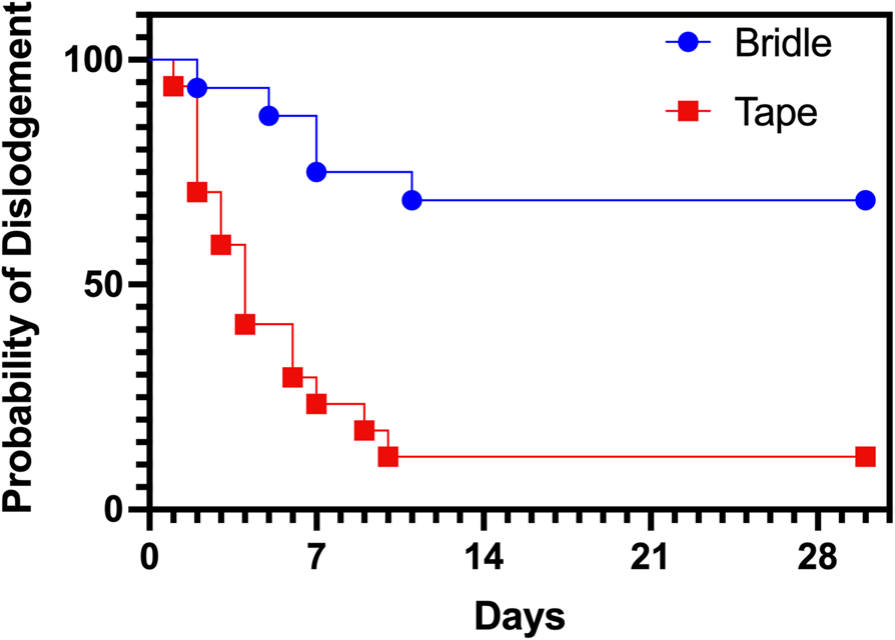
Kaplan–Meier analysis of nasoenteric feeding tube survival from placement to unintentional dislodgement

The primary analysis revealed the rate of feeding tube dislodgement was significantly higher in the tape group compared to the bridle group ((HR 6.3, 95% CI 2.38–16.5, *p* < 0.0001), with an attributable risk reduction of 57% and number needed to treat of 1.75. Please note that the median time to dislodgement could not be estimated in the bridle group because fewer than 50% of patients experienced the event during follow-up (Table 2). Kaplan–Meier analysis of tube survival illustrates these findings (Fig. 3). Dislodgement within 30 days occurred in 15 of 17 patients (88.2%) with tape-secured NG tubes and 5 of 16 patients (31.3%) with bridle-secured tubes. The risk of early dislodgement was significantly higher in the tape group (risk ratio = 2.82; 95% CI: 1.34–5.96; *p* = 0.001) (Table 2). Analyzing feeding tube dislodgement per tube-day as a complication, NG tube dislodgement occurred in 15 of 17 patients (88.2%) with tape securement and 4 of 16 patients (25.0%) with bridle securement within the first 10 days. Tape securement was associated with a significantly higher risk of early dislodgement, less than 10 days being of 3.4 (risk ratio = 3.5; 95% CI: 1.5–8.4; *p* < 0.0001). Lastly, an additional confirmation X-ray was required in 76% of tape patients versus 31% of bridle patients (RR = 2.45, 95% CI 1.13–5.30; *p* = 0.022). There was no difference in hospital length of stay, PICU length of stay, or minutes of missed nutrition between groups (Table 2). It is important to mention that missed nutrition time data was minimal; only three patients’ data were reported for tape compared to six patients for bridle group. Most patient resumed nutrition very quickly after tube dislodgement.

**Table 2:** Study outcomes

Outcome (*n*)	Tape	Bridle	Effect (95% CI)	*p* value
Time to dislodgment days median (IQR)	4 (2–8)	NA^b^ (11–30)	HR = 6.3^a^ (2.38–16.5)	< 0.001
Dislodgements *n*/*N* (%)	15/17 (88%)	5/16 (31%)	RR = 2.82 (1.34–5.96)	0.001
PICU LOS days (GM ± 95% CI)	16 (10–25)	20 (12–35)	RoGM = 0.76 (0.39–1.5)	0.413
Hospital LOS days (GM ± 95% CI)	24 (15–37)	38 (23–64)	RoGM = 0.62 (0.32–1.2)	0.140
Missed nutrition minutes median (IQR)	60 (60–86)	120 (60–120)	Δ = − 60 (− 60–15)	0.345
Extra placement X-rays *n*/*N* (%)	13/17 (77%)	5/16 (31%)	RR = 2.45 (1.13–5.30)	0.015

^a^Reference = bridle group, adjusted for age and gender

^b^NA—not available as median survival was not reached in the bridle group; curve truncated at 30 days, upper bound of IQR reflect censoring at 30 days (end of follow-up period)

*95% CI* 95% confidence interval, *RR* risk ratio, *IQR* interquartile range, *GM* geometric mean, *HR* hazard ratio, *RoGM* ratio of geometric means (data analyzed on the log (10) scale with Welch’s unequal-variance *t*; results back-transformed to give geometric means and ratios), *PICU* pediatric intensive care unit, *LOS* length of stay, Δ median difference

In the bridle group, none of the tube dislodgements was a result of mechanical bridle failure. The Bridle Pro® is designed to allow the feeding tube to slip through the device if tension is applied to the tube, which changes the internal diameter of the tube and allows it to slip through the bridle opening before causing damage to the nasal septum. Of the 5 unintentional tube dislodgements in the bridle group, there were 2 patients who dislodged the feeding tube in this manner and the bridle remained in place with no damage to the structure of the nasal cavity. The other 3 dislodgements were patients who vomited the tube out through the mouth with the bridle holding the tube securely in the nasal cavity. When comparing the tubes that were dislodged in each group, the tubes secured with the bridle also had a longer survival time, averaging 6.4 days to dislodgement compared to 4.3 days to dislodgement in the tape group although not statistically significant.

There were no serious adverse events although a few complications were noted from feeding tube securement including one in the bridle group and four in the tape group. There were no issues associated with bridle placement and one withdrawn due to allergic reaction mentioned above. There were four patients in the tape group who developed erythema and skin breakdown on the cheek where the tube was secured with tape. The erythema and breakdown resolved within several days after removal of the tape.

## Discussion

A nasogastric tube dislodgement can prevent the delivery of vital nutrition and medication to hospitalized children. A 2014 meta-analysis of studies in adults concluded that tubes secured with tape dislodged in 40% of placements, significantly higher than tubes secured with a bridle, which only dislodged in 14% of placements (*p* < 0.01) [5]. Lavoie et al. documented in a large retrospective study that children with bridled nasoenteric feeding tubes experienced fewer dislodgements, hospital days, ED encounters, and radiographic exposures than nasoenteric tubes secured with tape [7]. For the current cohort of 173 children with bridled NGTs, 62.4% (*N* = 108) went on to achieve full oral feeds and left the hospital, on average, 14 days earlier than children with unbridled NGTs, when accounting for clinically relevant predictors. Our study confirms and extends this finding using an open randomized controlled trial in hospitalized children.

To our knowledge, only one other randomized controlled study has evaluated the AMT Bridle Pro® in hospitalized children [11]. This study documented no difference in accidental dislodgement using the AMT Bridle Pro® (6 out of 21 dislodged) compared to standard practice (7 out of 22 dislodged). However, the authors commented on significant weakness including inconsistent study staff, change of AMT Bridle Pro® product design during the study period, and selection bias. The authors further document that due to inconsistent study staff not all patients who had an NG tube order were approached to be in the study resulting in selection bias. The study did document with staff surveys that the AMT Bridle Pro® system was easy to use with no difference in patient comfort or caregiver satisfaction. Our study improves on this previous study by approaching all patients who required an NG tube during the study period and following all patients for 30 days in which multiple dislodgements can occur. Furthermore, our study confirms and extends other studies that bridle securement is effective in increasing the consistent delivery of enteral feeds. It is known that consistent delivery of nutrition results in positive nutritional outcomes as well as maintaining the integrity of intestinal mucosa, reducing bacterial translocation, and decreasing infectious complications [12, 13].

Enteral nutrition is especially important in trauma patients with burns given the marked hypermetabolic response, the need to maintain the structural and functional integrity of the GI tract and support immune function [12, 14]. Our institution is a Level 1 Pediatric Trauma Center so several of the patients enrolled had suffered severe burns to the face, roughly 29% of the total population enrolled. However, feeding tubes are difficult to secure with burns to the face. The trauma care team noted to the investigators that use of the bridle in these patients prevented dislodgement and the material of the bridle was easier to clean than adhesive tape.

Previous studies have documented that the use of a nasal bridle can improve outcomes of cost and resource allocation in addition to nutrition [7, 15]. The authors chose to not perform a formal cost analysis as different sizes and brands of feeding tubes were used. Our findings suggest that feeding tubes may dislodge less frequently, potentially leading to reduced resource utilization. This could include fewer radiographs, replacement tubes, and staff hours spent on these procedures. However, further studies are needed to confirm these results and establish causation. Following the FDA clearance for the continuous use of the Bridle Pro®, the duration of the study was limited to 30 days. However, there are several reports and studies with longer-term use of a bridle device, some as long as 2–3 months [16]. In our study, there was only one patient in whom the bridle was intentionally removed because he had reached 30 days and still required the feeding tube.

There were several limitations to our study including no standardization of tape securement or reinforcement, no standardization of type of feeding tube, and having to stop the study early. For patients in the tape arm, the bedside nurse who placed the tube secured, reinforced, or replaced the tape as per the routine practice. No tube dislodgements occurred at the time of re-securement. Our study is limited by the lack of standardization in the taping method, which could introduce variability in the securement efficacy. This approach was chosen to mirror real-world clinical practices, enhancing the external validity of our findings. Nonetheless, future research could benefit from employing a standardized taping protocol to minimize variability and strengthen the comparison between securement methods. Once the bridle was properly placed, the device did not have to be changed or reinforced for the duration of the study.

Another limitation of the study was the variety of feeding tubes placed. The Bridle Pro® can be used with any brand of nasoenteric tube and for the purposes of this study, we used those stocked in our inpatient units. The bridles used for the study were either 5–6 Fr or 8–10 Fr and all tubes were either 6 Fr or 8 Fr. There were significantly fewer 5–6 Fr bridles used for this study as most of our patients were older and required an 8 Fr feeding tube. The brand of feeding tube was not collected as part of the study, which may contribute to different dislodgment rates. Lastly, the study was originally designed to recruit a total of 60 patients but stopped early by the authors because of the significant reduction in tube dislodgment in the bridle patients. This decision was done without an independent data safety monitoring board. Other factors that halted the study early was the lack of study staff due to COVID-19 pandemic. This low number of patients led to an additional limitation of a difference between groups of age and weight, although not significant. The authors suggest that the benefit of the bridle outweigh the risk of rare and infrequent adverse events that may be documented in a larger study.

Our immediate goal is to expand bridle use throughout the hospital and increase nursing and physician comfort with their use. Long-term goals would be to expand the use of the bridle to the outpatient setting and possibly send patients home with a nasoenteric feeding tube secured with a bridle. This may lead to fewer invasive gastrostomy tube procedures and improve family satisfaction with care.

## Conclusions

The AMT Bridle Pro® significantly decreases tube dislodgements in the securement of pediatric nasoenteric feeding tubes. Overall, the nasal bridle is an effective method to ensure a consistent delivery of nutrition and medications to hospitalized children.

## Supplementary Information

Supplementary Material 1.

## Data Availability

The datasets used and/or analyzed during the current study are available from the corresponding or first author on reasonable request.
